# Repeated intravenous transplantation of human umbilical cord mesenchymal stem cells does not promote tumorigenesis in EGFR-mutated lung cancer mice

**DOI:** 10.1093/stcltm/szae065

**Published:** 2025-09-02

**Authors:** Zepeng Zhang, Anhua Xu, Qian Zhou, Fei Wen, Fenghua Chen, Hansen Chen, Hu Wang, Liang Chen, Zhenyu Ju, Yuanlong Ge

**Affiliations:** Key Laboratory of Regenerative Medicine of Ministry of Education, Institute of Aging and Regenerative Medicine, College of Life Science and Technology, Jinan University, Guangzhou 510632, People’s Republic of China; Department of Geriatrics, Medical Center on Aging of Ruijin Hospital, Shanghai Jiao Tong University School of Medicine, Shanghai 200025, People’s Republic of China; Key Laboratory of Regenerative Medicine of Ministry of Education, Institute of Aging and Regenerative Medicine, College of Life Science and Technology, Jinan University, Guangzhou 510632, People’s Republic of China; MOE Key Laboratory of Tumor Molecular Biology and Key Laboratory of Functional Protein Research of Guangdong Higher Education Institutes, Institute of Life and Health Engineering, College of Life Science and Technology, Jinan University, Guangzhou 510632, People’s Republic of China; Key Laboratory of Regenerative Medicine of Ministry of Education, Institute of Aging and Regenerative Medicine, College of Life Science and Technology, Jinan University, Guangzhou 510632, People’s Republic of China; Key Laboratory of Regenerative Medicine of Ministry of Education, Institute of Aging and Regenerative Medicine, College of Life Science and Technology, Jinan University, Guangzhou 510632, People’s Republic of China; Key Laboratory of Regenerative Medicine of Ministry of Education, Institute of Aging and Regenerative Medicine, College of Life Science and Technology, Jinan University, Guangzhou 510632, People’s Republic of China; GCH Regenerative Medicine Group-Jinan University Joint Research and Development Center, Jinan University, Guangzhou 510632, People’s Republic of China; Key Laboratory of Regenerative Medicine of Ministry of Education, Institute of Aging and Regenerative Medicine, College of Life Science and Technology, Jinan University, Guangzhou 510632, People’s Republic of China; MOE Key Laboratory of Tumor Molecular Biology and Key Laboratory of Functional Protein Research of Guangdong Higher Education Institutes, Institute of Life and Health Engineering, College of Life Science and Technology, Jinan University, Guangzhou 510632, People’s Republic of China; Key Laboratory of Regenerative Medicine of Ministry of Education, Institute of Aging and Regenerative Medicine, College of Life Science and Technology, Jinan University, Guangzhou 510632, People’s Republic of China; Key Laboratory of Regenerative Medicine of Ministry of Education, Institute of Aging and Regenerative Medicine, College of Life Science and Technology, Jinan University, Guangzhou 510632, People’s Republic of China

**Keywords:** UC-MSC, xenograft, lung cancer, adenocarcinoma, EGFR

## Abstract

Mesenchymal stem cells (MSCs) are extensively studied in clinical trials for their potential therapeutic applications in degenerative and inflammatory diseases and disorders. Despite the lack of clinical evidence indicating that MSCs induce carcinogenesis, the immunosuppressive and proangiogenic functions of MSCs are considered as potential risks involving immune escape and tumor occurrence in programming tumor microenvironment. Previously, many groups had studied the tumorigenic safety of MSCs, but most of these studies were modeled in immuno-deficient mice with different types and sources of transplanted tumors, leaving varied and controversial conclusions. In this study, we developed a new xenograft model by repeatedly transplanting human umbilical cord mesenchymal stem cells (UC-MSCs) into transgenic mice via tail vein. These mice, carried a human-derived mutated *EGFR* with a normal immune system, were used to investigate whether UC-MSCs promote the occurrence of lung adenocarcinoma. The duration, dynamics, and pathological characteristics of the early stages of the disease were analyzed. In general, repeated transplantation of UC-MSCs neither accelerated the occurrence of lung cancer and the progression of bronchial alveolar carcinoma nor promoted a pro-tumor immune microenvironment. These results suggest that repeated transplantation of UC-MSCs does not increase the risk of lung cancer.

## Graphical Abstract

**Graphical Abstract:**
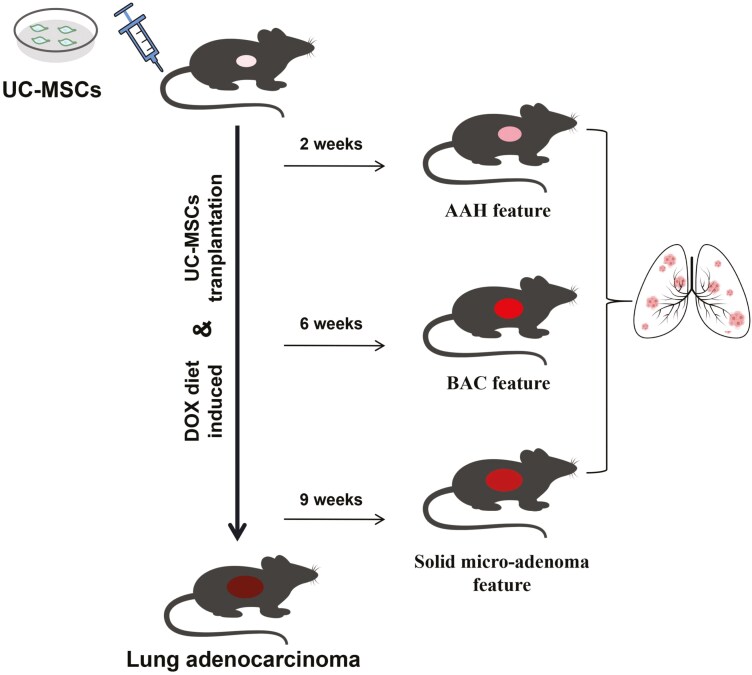

**Significance Statement**

Concerns about the potential lung tumorigenesis risk in intravenous transplantation of human umbilical cord mesenchymal stem cells (UC-MSCs) for stem cell-based therapy are due to their immune regulatory effects. Previous studies, modeling with immune-deficient mice and xenograft tumor, did not fully evaluate the impact of MSCs on carcinoma occurrence within the immune microenvironment. This study used a model with xenograft of UC-MSCs in inducible lung cancer mice with normal immune system. Our findings suggest that repeated UC-MSCs transplantation does not accelerate carcinoma initiation or development, indicating minimal tumorigenesis risk of intravenous UC-MSCs transplantation.

## Introduction

Mesenchymal stem cells (MSCs) are a kind of stem cells originating from multiple tissues, including bone marrow, adipose, umbilical cord, etc.^1^ with multipotency and abundant secretion of growth factors and cytokine. The clinical use of MSCs has emerged as a key player in cell therapy. By large-scale in vitro culture and standard tests, allografts provide a strategy with lower cost and security risks compared to autografts.^2^ Given that the immunogenicity of MSCs is variable depending on the sources and donors,^3^ tests for hemocompatibility and safety must be conducted to prevent adverse events such as thrombosis and embolism. Bone marrow MSCs (BM-MSCs) are the most commonly used source in clinical trials.^4^ Recently, due to the strong abilities in differentiation, migration, and secretion,^5^ and lower immunogenicity resulting from low expression of HLA-DR and MHC-I, and lack of MHC-II, umbilical cord MSCs (UC-MSCs) are drawing more attention to research and clinical trials.^6-8^ Derived from perinatal tissue, UC-MSCs offer noninvasive isolation, high extension potential, and genetic stability,^5,9,10^ and are being applied as a promising product in cell therapy for conditions such as premature ovarian failure,^11^ decompensated liver cirrhosis,^12^ type I diabetes^13^ and osteoarthritis.^14^

A large body of literatures and preclinical data support that the main modalities that benefit the recipients are according to the differentiation and immune regulatory through paracrine or phagocytic.^1,15^ The commonly used delivery methods for MSC products involve intravascular infusion or local tissue injection.^15^ Compared to the varied requirements of different injection locations, intravascular infusion is easier to operate and accounts for almost half the proportion in clinical trials.^16^ Instead of circulating in the blood, most of the MSCs transplanted intravascularly are trapped in the lung capillaries and eliminated by macrophages within a few days.^15-18^ This process, called efferocytosis, is evoked to explain the strong modulation in the local and systemic immune system following MSC infusion.^19,20^ Recently, more groups have reported that repeated transplantation of MSCs shows better therapeutic effects in immune dysregulation-related diseases.^13,21-23^ However, the microenvironment modified by MSCs often presents as immune suppressive, which is similar to what occurs during cancer development.^24-26^ Even though lack of evidence in carcinogenesis, careful study of the tumorigenic safety of MSC transplantation is still needed.

Every year, there are approximately 2 million new cases of lung cancer and 1.76 million deaths, making it one of the most commonly diagnosed cancers globally and the leading cause of cancer-related deaths.^27^ Since intravascular transplantation is a popular delivery method, the MSCs trapped in the lungs would directly interact with the microenvironment, and modulate the immune system in both enhancing and suppressing immune responses depending on the context. To evaluate whether these MSCs would promote the programming of the tumor microenvironment and increase the risks of lung cancer occurrence, many studies have been conducted. By coculturing human UC-MSCs or BM-MSCs with lung cancer cells in vitro, several groups have shown that MSCs inhibited invasion and proliferation, and even induced cellular apoptosis^28^ or inhibition of epithelial-mesenchymal transition.^29^ Cotransplanting BM-MSCs or MSCs-derived culture medium with lung adenocarcinoma cells subcutaneously could inhibit the growth of adenocarcinoma.^29^ When BM-MSCs were pretreated with TNF-α, the secretion of TRAIL and DKK3 inhibited tumor progression and induced apoptosis of lung tumors modeled by intravenous injection of breast cancer cells, which was not observed in untreated MSCs.^30^ On the other hand, some groups observed different results. Cotransplanting a mixture of human UC-MSCs with lung adenocarcinoma cells subcutaneously revealed an increase in tumor growth by inhibiting PTEN of cancer cells with extracellular vesicles.^31^ Several groups also found that transplantation of mouse BM-MSCs would promote the proliferation of subcutaneous tumors^32^ or lung metastasis modeled with lung carcinoma cells by suppressing the response of immune cells.^33^ So far, the tumorigenic safety of MSCs is still varied and controversial as the absence of strong clinical evidence and the research models compromised in mimicking the occurrence of lung carcinoma, which were modeled in immuno-deficient mice with xenografts of different kinds of cancer cells.

The *EGFR* mutation is the most common driver mutation in over 60% of non–small cell lung cancer (NSCLC) cases, which is a main pathological type of lung cancer.^34^ The partial deletion of the 19th exon, also known as *EGFR (Del19)*, occurs most frequently and is widely studied as a transgenic model for lung cancer research and therapy.^35,36^ In this study, we used a mouse model, *TetO-EGFR(Del19);CC10-rtTA*, by crossing transgenic mice *TetO-EGFR (Del19)* with *CC10-rtTA*. These mice specifically express a human-derived oncogene, 19th-exon-deleted *EGFR*, in alveolar epithelial cells in the presence of doxycycline (DOX), leading to lung adenocarcinoma.^37^ During the long time windows from the initiation of carcinoma to the development of adenoma,^35,36^ we performed repeated transplantation of UC-MSCs and characterized the dynamics through live imaging and pathological analysis. In general, frequently repeated transplantation of UC-MSCs neither accelerated the occurrence and progression in the early stage of lung cancer nor promoted a pro-tumor immune microenvironment or angiogenesis. Our results suggested that even in individuals with low tumorigenic burden, repeated transplantation of UC-MSCs barely increased the risk and promoted the early progression of lung cancer, thereby providing new evidence for the safety in the application of UC-MSCs. In addition, our study might provide a new model for evaluating the carcinogenesis of MSC products from different tissues and donors.

## Materials and methods

### Cell culture

The UC-MSCs used in this experiment were obtained from 3 different healthy donors from GCH Regenerative Medicine Group-Jinan University Joint Research and Development Center. The UC-MSCs were cultured in complete medium DME/F12 (Gibco, C11330500BT) supplemented with 10% fetal bovine serum (ExCell Biology, FND500) and 1% P/S (NCM, C100C5). The UC-MSCs used in this experiment were at passages 4 or 5 (P4 or P5). Cells were passaged when they reached 70%-80% confluence. Two days before transplantation, the UC-MSCs were switched to a serum-free medium (Nuwacell, RP02010-01). The cells were cultured at 37 °C with 5% CO_2_.

### Mice

*TetO-EGFR (Del19)* mice were crossed with *CC10-rtTA* mice to generate mice of the *TetO-EGFR (Del19);CC10-rtTA* genotype on a C57BL/6J background. Experiments were conducted using mice aged 2-3 months. To induce lung adenocarcinoma, mice were fed with DOX diet throughout the experiments. The DOX diet, which contained 0.1% DOX, was used in the majority of experiments, otherwise indicated with 0.2% DOX. Mice were housed under standard conditions and kept on a 12-hour light/dark cycle with ad libitum access to food and water. All animal procedures were conducted in strict accordance with guidelines for the care and use of laboratory animals, under the licence number: 20221026-15, as approved by the Institute of Laboratory Animal Science, Jinan University.

### Xenograft model by transplantation of UC-MSCs

When UC-MSCs reached around 70%-80% confluence, they were digested with trypsin (NCM, C100C1), and cells were then collected by centrifuging at 1200 rpm for 5 minutes at room temperature. The cell pellet was resuspended in PBS (eLGbio, EH80256) and passed through a 300 MeSH for counting. The cells were collected again by centrifugation and resuspended in PBS at a concentration of 5 million/mL. Each 1mL contains 30 U of sodium heparin (Sangon, A603261-0001). Before transplantation, the cells were passed through a 300 MeSH and each mouse was slowly transplanted with 100 μL of UC-MSCs or PBS via the tail vein. The EC mice were injected twice a week under the induction of a diet with 0.1% DOX in the majority of experiments, unless otherwise indicated.

### Immunohistochemistry and hematoxylin-eosin staining

After fixation with poly formaldehyde, the lung tissues underwent paraffin embedding, sectioning, and hematoxylin-eosin (H&E) staining, completed by Servicebio. The slices were baked at 55 °C for 2 hours and then rehydrated by immersion in xylene I, xylene II, anhydrous ethanol, 95% ethanol, 85% ethanol, and 75% ethanol for 5 minutes each. For immunohistochemistry (IHC), endogenous peroxidase was inactivated by incubating the slices in 3% hydrogen peroxide prepared with 0.1 M PBS at room temperature for 10 minutes. Antigen retrieval was performed using 0.1M citrate, boiling the slides in a pressure cooker for 10 minutes, and allowing them to cool down to room temperature naturally. The slices were then sealed with 10% GS (Thermo, 16210064) at room temperature for 1 hour and incubated with anti-Ki67 (Abcam, ab15580, 1:500) and anti-CD3 (ABclonal, A19017, 1:200) primary antibodies overnight at 4 °C. The next day, the slices were incubated with a rabbit secondary antibody (ZSGB-bio, PV-6001) at room temperature for 40 minutes and stained using a DAB staining kit (Boster, AR1022) for approximately 1 minute, stopping the reaction with tap water. The slices were then stained with hematoxylin at room temperature for 4 minutes, rinsed with tap water for 3 minutes, differentiated with 0.5% hydrochloric acid alcohol for 20 seconds, rinsed again with tap water for 3 minutes, and counterstained by immersion in 0.6% ammonia water for 30 seconds before rinsing with tap water. The slices were then dehydrated by immersion in 75% ethanol, 85% ethanol, 95% ethanol, anhydrous ethanol, xylene II, and xylene III for 5 minutes each before being sealed with neutral gum. The slides were scanned using a Pannoramic MIDI (3DHISTECH). Statistical analysis of the images was performed using SlideViewer or ImageJ software.

### Immunofluorescence

Slide hydration was performed as in IHC. Membrane permeabilization was carried out at room temperature for 10 minutes using 0.5% Triton-X100. The slides were sealed with 10% GS at room temperature for 1 hour and incubated with anti-F4/80 (CST, 30325S, 1:500) and anti-CD31 (Proteintech, 28083-1-AP, 1:2000) primary antibodies overnight at 4 °C. The next day, the slides were incubated with a fluorescent secondary antibody of rabbit (Jackson, 111-545-003, 1:300) at room temperature in the dark for 1.5 hours. An autofluorescence quenching kit (Vector, SP-8400) was used to remove nonspecific fluorescent signals. The sections were then sealed with Vector Laboratories (Vector, H-1500). After air drying in the dark, photographs were taken using a fluorescence microscope (ZEISS). Statistical analysis of the images was performed using ZEN or ImageJ software.

### Computed tomography imaging

To monitor tumor development in mice, computed tomography (CT) scans of the lungs were performed using a Pingseng Healthcare recorder SNC-100 every one or 2 weeks as indicated in the experiments. After anesthesia with isoflurane, each mouse was scanned through 600 layers of the whole chest. The results were reconstructed to obtain a set of images with a grayscale representation of the lungs. The results presented for each mouse were representative images with similar relative areas and locations.

## Results

### Intravenous injected UC-MSCs repeatedly to transgenic lung cancer mice with normal immune system in a xenograft model

**Figure 1.**
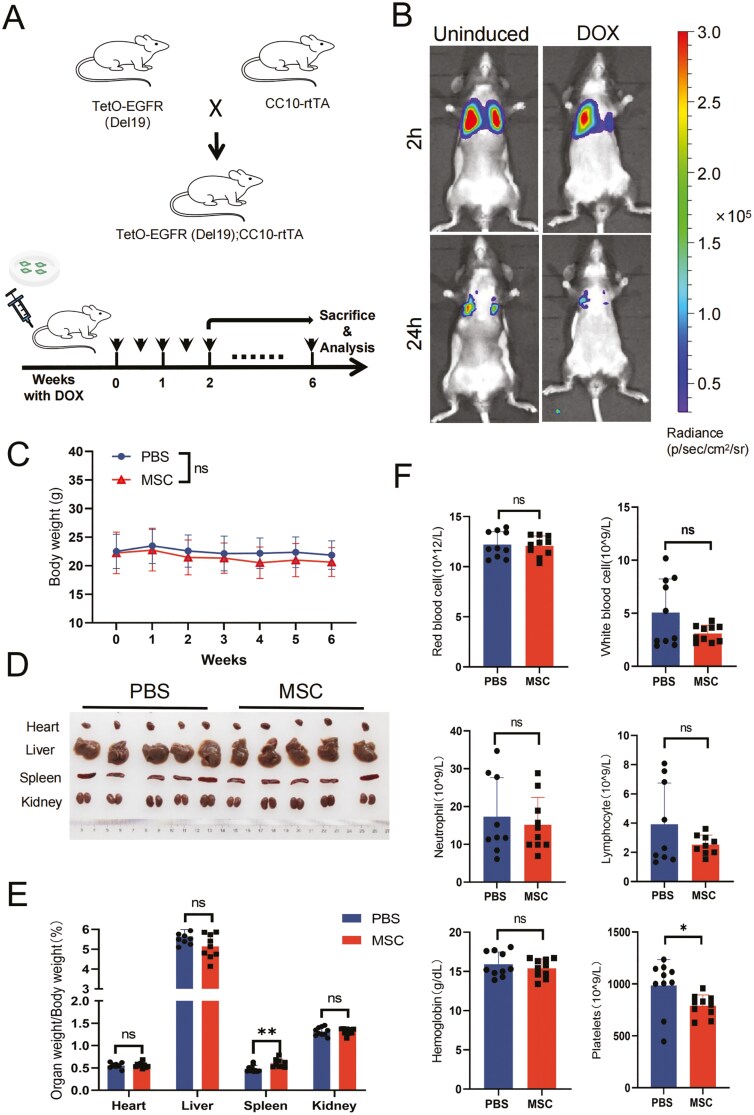
UC-MSCs xenograft model for evaluating tumorigenesis in EGFR (Del19) lung cancer mice. (A) Schematic of the xenograft model. *TetO-EGFR (Del19)* mice were crossed with *CC10-rtTA* mice to generate T*etO-EGFR (Del19);CC10-rtTA* mice (designated EC mice). Lung cancer was induced in these mice with a DOX diet. UC-MSCs were transplanted via the vein twice weekly, with PBS injected as a control. CT scans and weight records were taken weekly. Lung cancer-bearing mice were studied after the second and sixth weeks of induction. (B) Transplantation of 0.5 million luci-MSCs via the tail vein resulted in accumulation of luci-MSCs in lung tissue, which were cleared within days. (C) Body weight of mice in the MSCs and PBS groups. Data are presented as mean ± SD; *n* = 10 for each group, 2-way ANOVA. (D) Representative images of mice hearts, livers, spleens, and kidneys after 6 weeks of DOX diet induction. (E) Statistical analysis of mice heart, liver, spleen, and kidney weight relative to body weight. Data are presented as mean ± SD, *n* = 10 for each group, *t* test. (F) Peripheral blood analysis showing the number of red blood cells, white blood cells, neutrophils, lymphocytes, and hemoglobin, platelets content. Data are presented as mean ± SD; *n* = 10 for each group, *t* test. *P < .05.

The human UC-MSCs were confirmed based on the criteria defined by the International Society for Cellular Therapy.^38^ After being cultured for several passages, the morphology of UC-MSCs (P4 or P5) was observed and several classical surface markers were characterized by flow cytometry analysis (Supplementary Figure S1A, S1B). By cocultured with RAW264.7 and HUVEC, the immunomodulatory and angiogenic capabilities of UC-MSCs were validated (Supplementary Figure S1C-S1J), consistently with previous studies. To evaluate whether UC-MSCs affect the occurrence or increase the risk of lung cancer, an induced lung cancer mouse model, *TetO-EGFR(Del19);CC10-rtTA* (designated EC mice), was used by crossing 2 transgenic strains of C57B/6 mice carrying *TetO-EGFR(Del19)* and *CC10-rtTA* (Figure 1A).^39^ In the presence of DOX, the rtTA fusion proteins bind with DOX to form an allosteric complex that can efficiently bind to the TetO promoter to initiate transcription of the mutated *EGFR* (Supplementary Figure S2A), leading to gradual carcinogenesis of the alveolar epithelium and initiation of lung adenocarcinoma with bronchial alveolar carcinoma features.^39^ While inducing lung cancer in EC mice by feeding a diet with DOX, the cultured early passage UC-MSCs were continuously injected (Figure 1A), and the process of carcinoma was monitored by CT scanning on SNC-100 (Supplementary Figure S2D). To confirm the distribution of UC-MSCs after carcinogenesis, luci-MSCs, which are UC-MSCs infected by a lentivirus carrying luciferase, were injected into the vein, followed by the intraperitoneal injection of d-luciferin, and the bioluminescence of luci-MSCs in vivo was imaged by an IVIS system. The majority of luci-MSCs concentrated in the lungs during the occurrence of carcinoma (Supplementary Figure S2B-S2D), and decreased dramatically within days (Figure 1B), which is consistent with a previous study. After 6 weeks of induction, the body weight of UC-MSCs transplantation mice (the MSC group) showed no difference compared to the control group (the PBS group), which was injected with the same volume of PBS instead of cell suspension (Figure 1C). Images of major organs were captured, and the weight was calculated as a relative ratio to body weight (Figure 1D, 1E). No significant changes were observed in the relative weight of hearts, livers, and kidneys. The spleens were slightly enlarged, which might be due to the immune response, as has also been reported clinically.^40^ We also performed an analysis of the main components of peripheral blood (Figure 1F). Most indices, such as the count (per liter) of red blood cells, white blood cells, lymphocytes, neutrophils, and the content of hemoglobin, were similar between the MSC and PBS groups, while the platelets slightly decreased (Figure 1F). As the spleen performs the clearance of platelets, the decrease in platelets might be triggered by the enlarged spleen. In general, our xenograft did not induce obvious adverse effects in EC mice.

### Occurrence of carcinoma did not accelerate when transplanted UC-MSCs repeatedly

**Figure 2.**
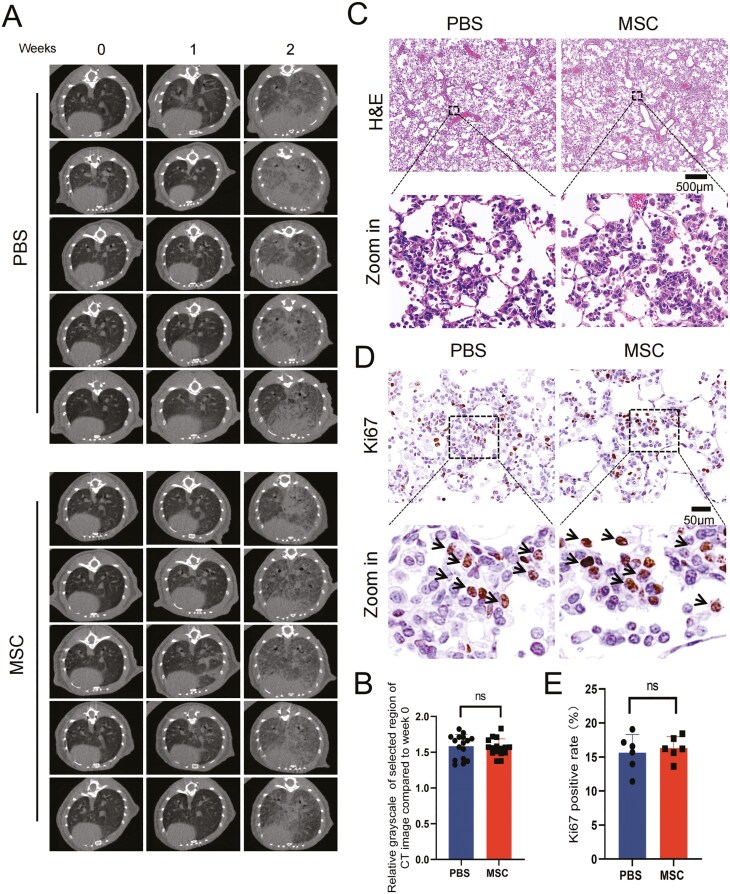
UC-MSCs transplantation did not accelerate the occurrence of carcinoma. (A) Representative CT scan images at different time points, 2 weeks after induction with a DOX diet. (B) Grayscale statistical analysis of CT scans 2 weeks post-DOX diet induction. The relative grayscale of selected regions in the CT images was normalized to week 0. Data are presented as mean ± SD, *n* = 16 for each group, *t* test. (C) Representative images of H&E stained lung tissue 2 weeks post-DOX diet induction, scale bar = 500 μm. (D) Representative images of IHC withKi67 staining for lung tissue 2 weeks post-DOX diet induction. Ki67-positive cells are indicated by blackarrows in the enlarged images, scale bar = 50 μm. (E) Statistical analysis of the rate of Ki67-positive cells in (D). Data are presented as mean ± SD, *n* = 6 for each group, *t* test.

In a previous study, a dramatic elevation of mutated *EGFR* at the protein level was detected after 2 weeks of DOX induction in EC mice, and the early precancerous atypical adenomatous hyperplasia (AAH) lesions started to appear.^39^ To investigate whether UC-MSCs promote the occurrence of carcinoma, lung density was monitored by CT scanning at indicated time points since the EC mice were fed a diet with DOX (Figures 1A, 2A). By analyzing the reconstruction results of the CT scans, we found that the density increased in the majority of the mice after 2 weeks (Figure 2B), which represents the appearance of AAH, consistent with previous research.^39^ To evaluate the burden of cancer, the density of the lung in the images was quantified and normalized to each mouse before induction (Figure 2C). The grayscale analysis showed that the relative density was similar between the PBS group and the MSC group transplantation. To further determine the AAH of the mice, the mice were sacrificed after 2 weeks of induction, lung tissues were fixed and embedded in paraffin for slicing and staining. The early precancerous AAH and the increase of immune cell infiltration were observed by H&E staining (Figure 2D), which are regarded as the typical characteristics of the occurrence of lung cancer. No difference in AAH was shown in the MSC group and the PBS group (Figure 2D). As the proliferated cells are characterized by high expression of Ki67, which is regarded as a marker of dysplasia of cancer cells, we performed IHC staining of Ki67 on lung tissue sections (Figure 2E), and statistical analysis of Ki67 positive cells (Figure 2F), showing that repeated transplantation of UC-MSCs for 2 weeks would not increase the ratio of proliferating alveolar epithelial cells (Figure 2F). These results indicated that repeated transplantation of UC-MSCs for 2 weeks did not accelerate the early precancerous AAH, suggesting that the transplantation of UC-MSCs would not promote the occurrence of lung cancer.

### Immune microenvironment at lung tissues during occurrence of carcinoma

**Figure 3.**
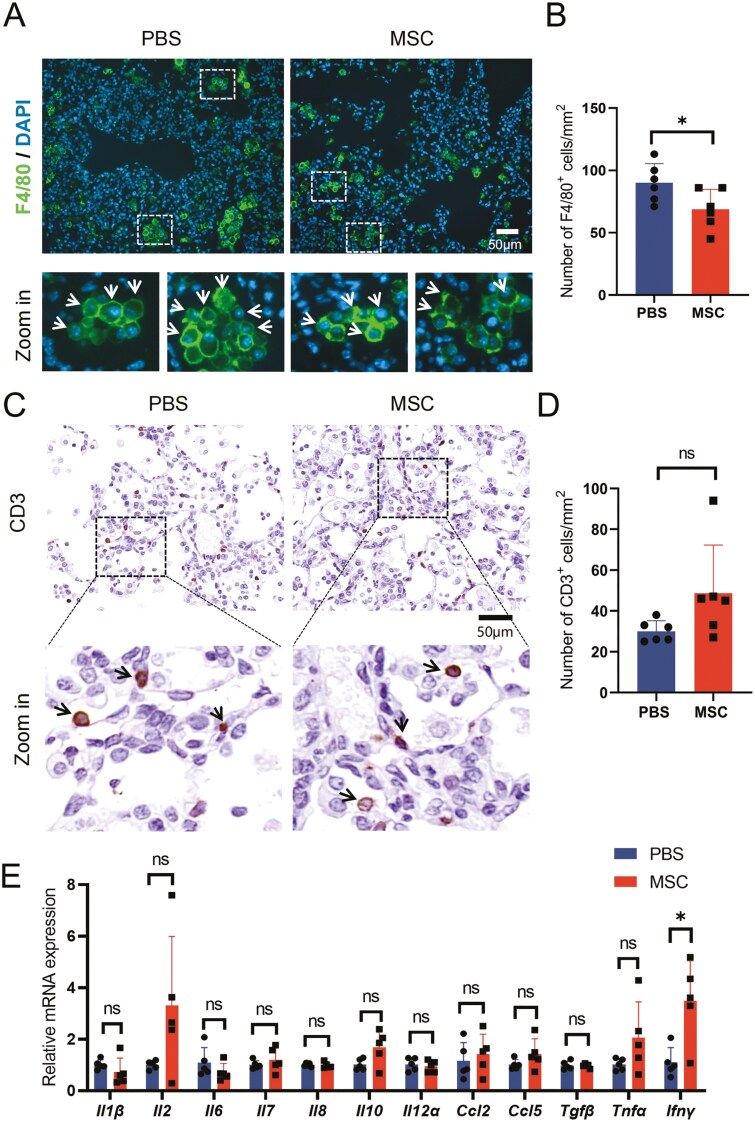
Infiltration of immune cells during occurrence of carcinoma. (A) Representative images of immunofluorescence with F4/80 staining for lung tissue 2 weeks post-DOX diet induction. F4/80-positive cells are indicated by white arrows in the enlarged images, scale bar = 50 μm. (B) Statistical analysis of F4/80-positive cells in (A). Data are presented as mean ± SD, *n* = 6 for each group, *t* test. (C) Representative images of IHC with CD3 staining for lung tissue 2 weeks post-DOX diet induction. CD3-positive cells are indicated by black arrows in the enlarged images, scale bar = 50 μm. (D) Statistical analysis of CD3-positive cells in (C). Data are presented as mean ± SD, *n* = 6 for each group, *t* test. (E) Relative expression of the indicated gene in lung tissue from the MSC and PBS groups 2 weeks postinduction. Data are presented as mean ± SD, *n* = 5 for each group, *t* test. *P < .05.

The microenvironment provides an important niche for the occurrence and development of lung adenocarcinoma, among which inflammation-related cells and mediators are key components in reprogramming the tumor microenvironment.^41^ MSCs can regulate the polarization of macrophages and the activation of T cells through interaction or secretion.^42^ As an important component of the innate immune system, macrophages are involved in immune defense, regulation, surveillance, and many other processes in vivo.^43^ During the occurrence of carcinoma, macrophages can not only phagocytose cancer cells but also recruit cytotoxic T cells to kill the cancer cells through antigen presentation and secretion of inflammatory factors.^44^ Previous studies showed that MSCs could promote M2 polarization of macrophages through efferocytosis or paracrine factors,^17,33^ which may create an immune-suppressive microenvironment and inhibit the recruitment of effector T cells.^44^ To investigate the effects of UC-MSCs on the immune microenvironment during the occurrence of AAH, we first detected the infiltration of macrophages in lung tissue by staining anti-F4/80 on lung tissue sections (Figure 3A), which is an important marker of macrophages, and quantified the cell density in images (Figure 3B). The results showed that UC-MSCs transplantation for 2 weeks slightly reduced macrophage infiltration (Figure 3B). Then, we detected the infiltration of T cells in lung tissue by anti-CD3 staining (Figure 3C), and quantified the cell density in images (Figure 3D). Interestingly, the infiltration of T cells in lung tissue seemed to slightly increase, but not significantly (Figure 3D). To evaluate the inflammatory level of lung tissue, the transcription of inflammatory factors and chemokines was analyzed by Q-PCR (Figure 3E). Most of these gene expressions did not change (Figure 3E). The increase in *Ifnγ* may be the T-cell response to the early occurrence of AAH.^45^ In brief, these results suggest that repeated transplantation of UC-MSCs would not affect the immune microenvironment during early precancerous AAH.

### Repeated transplantation of UC-MSCs barely affects the dysplasia of bronchial alveolar carcinoma and the formation of solid micro-adenocarcinomas

**Figure 4.**
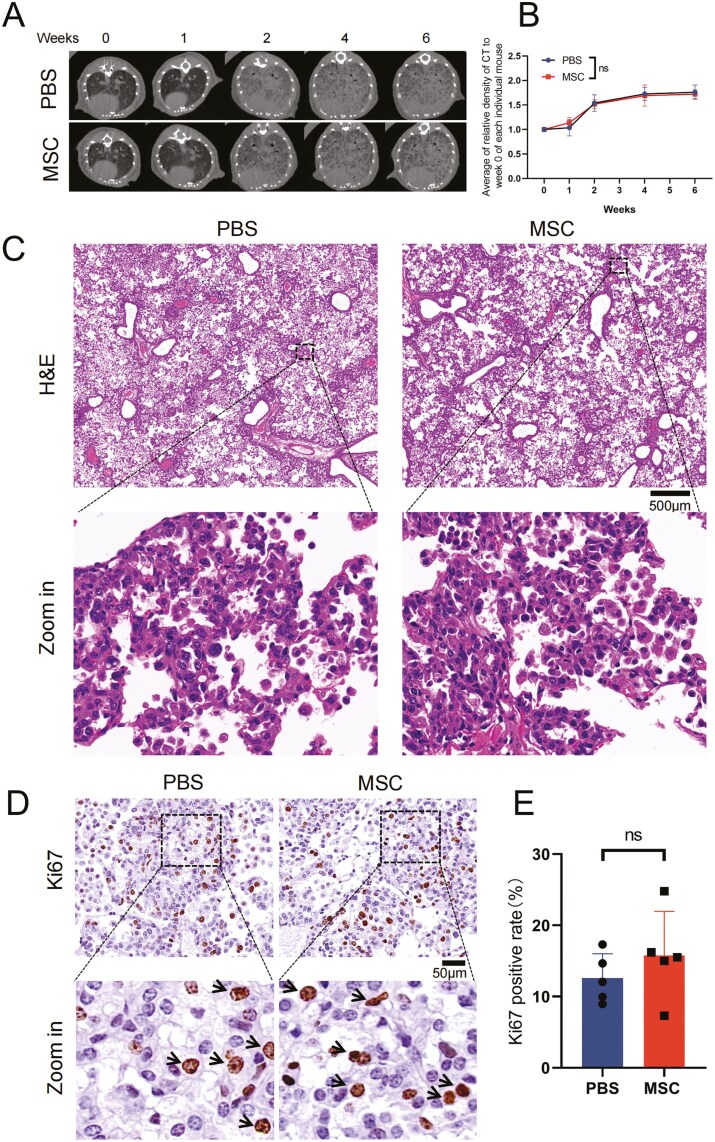
Repeated transplantation of UC-MSCs did not aggravate the dysplasia of BAC. (A) Representative CT scan images at different time points, 6 weeks after induction with a DOX diet. (B) Relative grayscale analysis of CT images from (A). The relative grayscale of selected regions in the CT images was compared to week 0. Data are presented as mean ± SD, *n* = 10 for each group, 2-way ANOVA. (C) Representative images of H&E stained lung tissue 6 weeks post-DOX diet induction, scale bar = 500 μm. (D) Representative images of IHC with Ki67 staining for lung tissue 6 weeks post-DOX diet induction. Ki67-positive cells are indicated by black arrows in the enlarged images, scale bar = 50 μm. (E) Statistical analysis of the rate of Ki67-positive cells in (D). Data are presented as mean ± SD, *n* = 5 for each group, *t* test. *P < .05.

A previous study showed that in our EC mice, the progression from the occurrence of carcinoma to the development of lung adenocarcinoma requires weeks of DOX induction. This process, referred to as AAH, appears in the early stage of continuous DOX induction, then multiple tumor foci of bronchiolar alveolar carcinoma (BAC) are present in the lungs, diffusely involving the parenchyma and many surrounding regions after 5-6 weeks.^39^ As our results suggested that the transplantation of UC-MSCs would not raise the risk or accelerate the occurrence of AAH, to further investigate whether UC-MSCs influence the development of lung adenocarcinoma, we continued to monitor the dynamics of lung density in EC mice during the dysplasia of BAC (Figure 4A). Reconstruction results of CT scanning showed that lung density significantly increased during the dysplasia of BAC compared with AAH (Figure 4B). The burden of cancer was quantified, showing that dysplasia seemed to stabilize after 4 weeks of induction (Figure 4C). Similar relative density and tendency were observed in the MSC group and the PBS group (Figure 4C). To confirm the cancer burden, the EC mice were sacrificed after 6 weeks of induction, lung tissues were fixed and embedded in paraffin for H&E staining, as shown in the figures (Figure 4D). Multiple tumor foci of BAC were present in the lungs of EC mice (Figure 4D). Consistent with the CT scanning results, the MSC group showed a similar level of BAC dysplasia compared with the PBS group. Furthermore, we also stained the Ki67 positive cells (Figure 4E) and quantified the Ki67 positive rate (Figure 4F), to determine the proliferation of alveolar epithelial cells. The results showed that repeated transplantation of UC-MSCs neither promoted the proliferation of alveolar epithelial cells nor aggravated the dysplasia of BAC in EC mice.

In addition, a previous study showed that after the initiation and dysplasia of carcinoma, invasive adenocarcinomas and solid features were present in EC mice around 8-10 weeks of induction.^39^ We also induced EC mice to the stage with solid adenocarcinomas by 9 weeks with DOX diet (0.1% and 0.2%). The CT scans were performed weekly, and the construction and quantified results showed that the tendency of cancer burden was similar in the MSC and PBS groups (Supplementary Figure S3A, S3B). The body weight and relative weight of the major organs of the mice showed no difference (Supplementary Figure S3C, S3D). When the mice were sacrificed, the lung tissue was fixed and embedded in paraffin for H&E staining (Supplementary Figure S3E). It can be clearly seen that both the UC-MSCs transplantation group and the PBS group had formed lung adenocarcinomas with solid features (Supplementary Figure S3E). We quantified the number of adenomas from the same area in the image of each slice (Supplementary Figure S3F), and also analyzed adenoma sizes (Supplementary Figure S3G). The number density and the size distribution of adenocarcinomas were similar in the 2 groups. Furthermore, the lung slices were subjected to Ki67 IHC staining and the rate of Ki67 positive cells was quantified (Supplementary Figure S3H, S3I), showing that repeated transplantation of UC-MSCs did not increase the number of proliferating alveolar epithelial cells. In summary, UC-MSCs transplantation would not promote the formation of solid micro-adenocarcinomas during the development of lung adenocarcinoma.

### The effect of UC-MSCs transplantation to immune microenvironment during the initiation to development of lung adenocarcinoma

**Figure 5.**
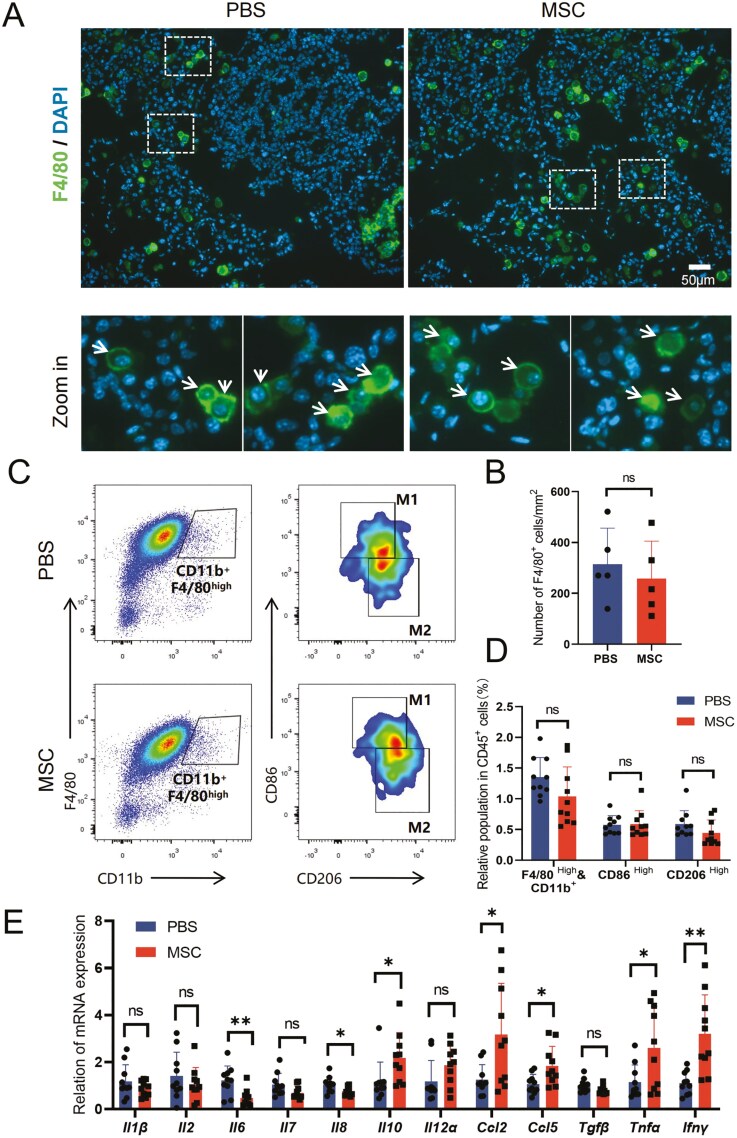
The macrophage showed no difference after repeated transplantation of UC-MSCs during the dysplasia of BAC. (A) Representative images of immunofluorescence with F4/80 staining for lung tissue 6 weeks post-DOX diet induction. F4/80-positive cells are indicated by white arrows in the enlarged images, scale bar = 50 μm. (B) Statistical analysis of F4/80-positive cells in (A). Data are presented as mean ± SD, *n* = 5 for each group, *t* test. (C) Representative FACS plots of macrophages (CD11b^−^ and F4/80^high^) from mouse lung tissue. The CD86^high^ and CD206^high^ gates from the macrophages represent the M1 and M2 populations, respectively. (D) Statistical analysis of macrophages (CD11b^−^ and F4/80^high^), M1 (CD86^high^), and M2 (CD206^high^) in (C). Data are presented as mean ± SD, *n* = 10 for each group, *t* test. (E) Relative expression of the indicated gene in lung tissue from the MSC and PBS groups 6 weeks postinduction. Data are presented as mean ± SD, *n* = 10 for each group, *t* test. *P < .05, **P < .01.

**Figure 6.**
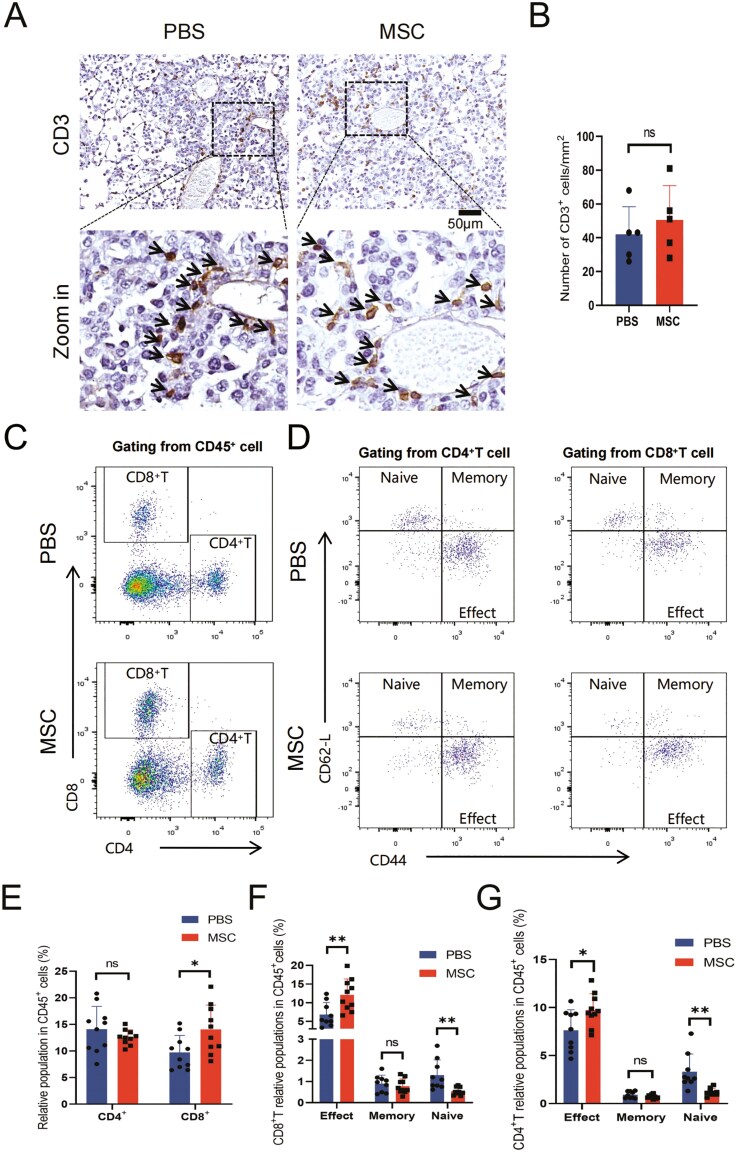
The effector T cells increased after repeated transplantation of UC-MSCs during the dysplasia of BAC. (A) Representative images of IHC with CD3 staining for lung tissue 6 weeks post-DOX diet induction. CD3-positive cells are indicated by black arrows in the enlarged images, scale bar = 50 μm (B) Statistical analysis of CD3-positive cells in (A). Data are presented as mean ± SD, *n* = 5 for each group, *t* test. (C) Representative FACS plots of CD4^+^ and CD8^+^ T cells from mouse lung tissue. (D) Representative FACS plots of naïve (CD44^−^, CD62-L^+^), memory (CD44^+^, CD62-L^+^), and effector (CD44^+^, CD62-L^−^) T cell groups from CD4^+^ or CD8^+^ T cells in mouse lung tissue. (E) Statistical analysis of CD4^+^ and CD8^+^ T cells in (C). Data are presented as mean ± SD, *n* = 10 for each group, *t* test. (F) Statistical analysis of naïve, memory, and effector T cells in (D). Data are presented as mean ± SD, *n* = 10 for each group, *t* test. (G) Statistical analysis of naïve, memory, and effector T cells in (D). Data are presented as mean ± SD, *n* = 10 for each group, *t* test. *P < .05, **P < .01.

The initiation of carcinomas to the formation of solid tumors is always coupled with disruption of homeostasis in the immunological microenvironment, although the immune system senses and constrains the cancer cells in the early stage.^46^ To determine whether UC-MSCs transplantation altered the reprogramming of the tumor microenvironment (TME), paraffin sections of lung tissue from 6 weeks induction EC mice were stained to evaluate the infiltration of macrophages (Figure 5A) and T cells (Figure 6A). Compared with 2 weeks of induction, the infiltrated macrophages increased approximately 2 times during the dysplasia of BAC (Figures 3B, 5B), while the infiltrated T cells increased less than 30% (Figures 3D, 6B). The total density number was comparable between the MSC and PBS groups (Figures 5B, 6B), as were the similar results observed in the EC mice after 9 weeks of induction (Supplementary Figure S4C, S4D). The polarization is associated with the different functions and roles of macrophages in the TME.^46,47^ The canonical classification of macrophages is referred to as M1 and M2 macrophages, which represent immune activation or suppression in the TME respectively.^48^ Although more studies have revealed complex mechanisms in the function and interaction of macrophages, the binary states are still used in most working model analysis.^49^ To detect the polarization of infiltrated macrophages, we prepared the cell suspension of lung tissue and performed flow cytometry to analyze the classical surface marker of macrophages (Figure 5C; Supplementary Figure S6A). The macrophages were marked by CD11b^+^ and F4/80^high^ from the CD45^+^ population, and the M1 and M2 subgroups were gated through CD86 and CD206 (Figure 5C). The number and ratio of M1 and M2 macrophages were similar between the MSC and PBS groups (Figure 5D), indicating that the polarization was not affected by repeated transplantation of UC-MSCs.

The infiltrated T cells in the TME always contain CD4^+^ and CD8^+^ T cells. The CD8^+^T cells are the main effector cells which function as cytotoxic cells, while CD4^+^T cells influence a variety of other immune cells as helper cells or regulatory cells.^50^ The recruitment and differentiation of different T-cell populations co-evolve with the TME. To confirm whether UC-MSCs transplantation altered the population, we also analyzed the infiltrated T cells by flow cytometry (Figure 6C; Supplementary Figure S7A). In the MSC group, the ratio of CD4^+^ cells in the CD45^+^ population showed no difference compared with the PBS group, while the CD8^+^ cell population increased (Figure 6C, 6D). Further, we investigated the subgroups of CD4^+^ and CD8^+^ cells by determining CD62-L and CD44 (Supplementary Figure S7A). In the MSC group, the CD8^+^ effector cells increased, and the naïve cells decreased (Figure 6D, 6G). Interestingly, although the CD4^+^ population showed no difference in both groups, the subpopulation was altered similarly to CD8, showing an increase of effector cells and a decrease of naïve cells (Figure 6D, 6F). Both CD4^+^ and CD8^+^ memory cells did not change in the 2 groups (Figure 6D, 6F, 6G).

In addition, we also detected the transcription of inflammatory factors to evaluate the immune microenvironment (Figure 5E; Supplementary Figure S4E). The alteration of inflammatory gene expression varied during the dysplasia of BAC. Similar to AAH, the *Ifnγ* expression was continuously higher in the MSC group (Figure 5E). The *Il10*, *Ccl2*, *Ccl5*, and *Tnfα* increased while the *Il6* and *Il8* decreased (Figure 5E). The high level of *Ifnγ* and *Tnfα* act as bidirectional roles in the formation of the TME.^51-53^ The *Ccl2* and *Ccl5* are always regarded as suppressive chemokines which are involved in the formation of the TME.^54,55^ On the other hand, a decrease of *Il6* and *Il8* in the tumor microenvironment is beneficial for the suppression of tumor proliferation.^56^ Nevertheless, these differences gradually reduced when the solid adenocarcinoma formed (Supplementary Figure S4E). Generally, even though changes were observed in subgroups of immune cells and the expression of inflammatory genes altered variably during the dysplasia of BAC, the immune microenvironment did not tend to be more malignant in the MSC group at the early stage of adenocarcinoma.

### The local angiogenesis did not change with repeatedly transplanted UC-MSCs

**Figure 7.**
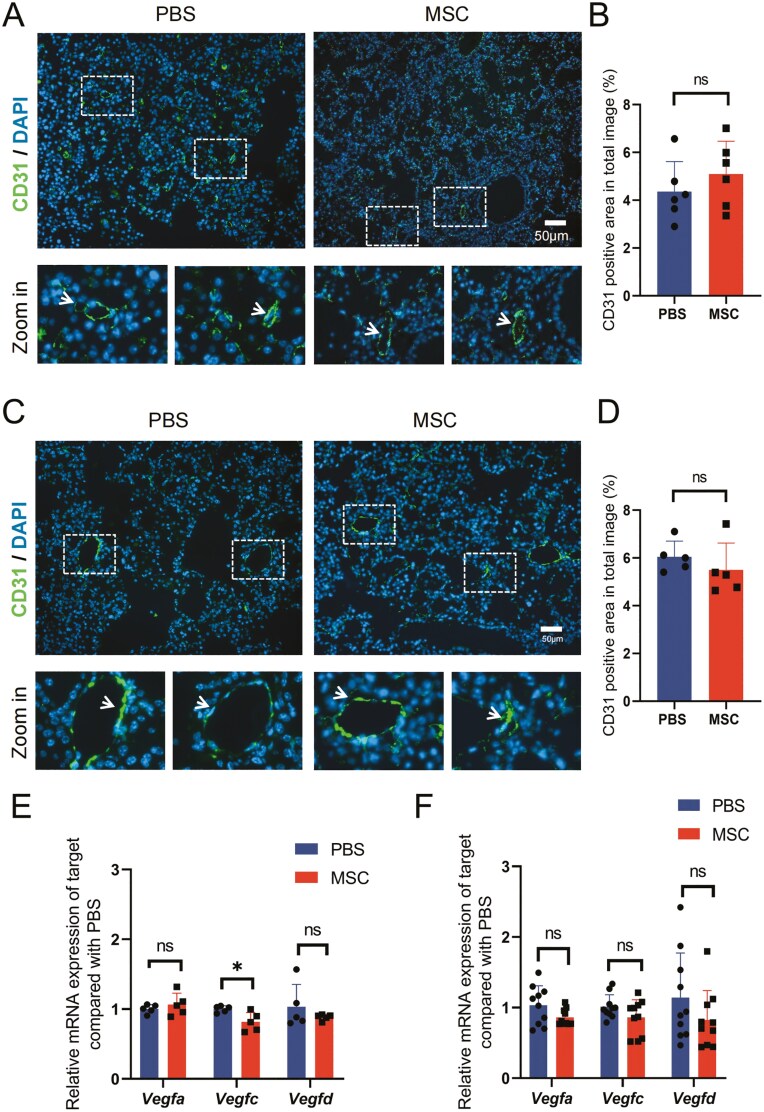
Angiogenesis show minor difference between MSCs and PBS groups. (A) Representative images of immunofluorescence (IF) with CD31 staining for lung tissue 2 weeks post-DOX diet induction. CD31-positive areas are indicated by white arrows in the enlarged images, scale bar = 50 μm. (B) Statistical analysis of the positive area in (A). Data are presented as mean ± SD, *n* = 6 for each group, *t* test. (C) Representative images of IF with CD31 staining for lung tissue 6 weeks post-DOX diet induction. CD31-positive areas are indicated by white arrows in the enlarged images, scale bar = 50 μm. (D) Statistical analysis of the positive area in (C). Data are presented as mean ± SD, *n* = 5 for each group, *t* test. (E) and (F) Relative expression of gene indicated of lung tissue in MSCs and PBS groups after 2 and 6 weeks induction, data are mean ± SD, *n* = 5 for each group, *t* test. *P < .05.

Angiogenesis, the process of new vessel development, is always present during tumorigenesis to maintain its supplement of nutrients and oxygen.^57^ Previous studies showed that MSCs could impact angiogenesis in multiple ways, such as secretion of growth factors and differentiation of related cells, which may contribute to tumor metastasis.^58,59^ To evaluate whether transplantation impacts angiogenesis during the initiation and development of lung cancer, we detected the vascular density by staining anti-CD31 of the lung tissue sections from the 3 stages in our study (Figure 7A, 7C; Supplementary Figure S5A). By quantifying the CD31 positive area, the relative area represented the vascular density was shown (Figure 7B, 7D; Supplementary Figure S5B). The vascular density increased from the AAH to BAC, then remained at a comparable level even until the formation of solid micro-adenocarcinomas (Figure 7B, 7D; Supplementary Figure S5B). At the same time, we also detected the expression of the vascular endothelial growth factor in lung tissues (Figure 7E, 7F; Supplementary Figure S5C). The vascular density and expression of *Vegf* were similar between the MSC and PBS groups, indicating that angiogenesis was not altered by UC-MSCs transplantation in EC mice.

## Discussion

MSCs are considered with great promise and are applied in clinical trials due to their ability for immunomodulation and tissue regeneration.^15,60^ Since allograft provides an efficient strategy for producing standardized, well-tested, low-cost clinical products,^2^ the study of the characteristics of different sources of MSCs and the strict quality control of donors are important and essential. UC-MSCs, a source from perinatal tissue, have been receiving increasing attention and are being investigated in clinical trials due to their features such as a low mutation rate and immunogenicity, low risk of tumorigenesis, high proliferation ability, and noninvasive origin.^60-63^ However, since many applications are in people with inflammatory dysregulation-associated diseases or disorders, or in aged people with aging immune system, the risks of whether the immunomodulation ability of MSCs is involved in the reprogramming of the TME and impacts the occurrence or early-stage development of carcinoma, need to be carefully investigated. In past years, many studies from different groups have given controversial conclusions on the promotion^64,65^ or inhibition^66,67^ of tumorigenesis by MSCs, which may vary in xenograft models with a variety of sources of cancer cells and MSCs. In our study, we used EC mice with normal immune system to induce cancer, which allowed us to observe the occurrence of lung adenocarcinoma in situ under the interaction with immune microenvironment. In contrast the xenograft of millions of cancer cells could not be regarded as the initiation of carcinoma to evaluate the risk, moreover, the deficiency of the immune system leads to a quite different niche. To maintain a longer resident time and reduce the impact of the instant blood-mediated inflammatory reaction, the cryostorage-derived early passaged UC-MSCs were thawed and cultured for days before intravenous injection.^15,16,60,68^ A previous study showed that the occurrence and early development of carcinoma in EC mice, which lasted for weeks of time window, contained 2 classical stages before the formation of solid micro-adenocarcinoma, namely AAH and BAC.^39^ During the induction of these stages, the cancer burden was characterized by CT scanning and pathological section, showed that repeated transplantation of UC-MSCs neither accelerated the occurrence of carcinoma nor promoted the dysplasia of cancer cells (Figures 2A-2E, 4A-4E), suggesting that UC-MSCs transplantation would not raise the risk of tumorigenesis.

Unlike the xenograft of MSCs in immune-deficient mice^69,70^ or the allograft model, the half-life of MSCs in mice with a normal immune system is shorter to days instead of weeks, which were estimated by immune cells, especially administrated by intravascular injection.^19,71,72^ Nevertheless, the half-life of BM-MSCs transplanted between mice of different backgrounds or repeatedly injected was much shorter due to the immune response.^73,74^ Similarly, even though there is a lack of sufficient clinical evidence, the limited tracing result and adverse events in hemocompatibility^75,76^ suggested that MSCs injected intravenously would not remain long by allograft due to the more complex genetic background in humans than in experimental animals. This may also suggest that xenograft MSCs provide a better understanding in the ways and mechanisms to impact as clinical products. It is worth noting that repeated transplantation of MSCs is regarded as a tendency for clinical application nowadays due to the better effect of therapy in several clinical trials.^13,77^ In the meantime, the continuous alteration of systematic or local immunity may compromise the immune cells gradually since the patient may suffer from immune dysregulation disorders, which leaves a higher risk in the promotion of the TME. In our study, to prolong the total time of MSCs staying in the lungs and mimic a more severe condition under a much higher transplantation frequency compared with clinical trials for evaluating safety, we performed repeated transplantation compensatorily. In this case, the mild alteration in groups of immune cells and expression of inflammatory genes (Supplementary Figure S4A-S4E) in the MSC group did not seem to promote a more malignant TME during AAH and BAC compared with the PBS group (Figures 3A-3E, 5A-5E, 6A-6G). The elevation of gene expression such as *Ifnγ*, *Tnfα*, and *Il10* (Figure 5E) could enhance the cytotoxic effect on tissue,^78^ which is consistent with the increase of the CD8^+^ T effector cell group (Figure 6E-6G). While the increase in *Ccl2* and *Ccl5* may recruit more macrophages to program the TME,^48,54,55^ the infiltration and polarization of macrophages were not changed (Figure 5A-5D). However, even though we did not test the development of lung adenocarcinoma after the formation of solid micro-adenocarcinoma, the local inflammation, as indicated by the continuous high level of *Ifnγ*, *Ccl2*, and *Ccl5*, suggests that the risk of TME reprogramming with MSCs may increase after the solid tumor appears with the exhaustion of immune cells. In addition, the advents of embolism increased during the early development of solid micro-adenocarcinoma (data not shown), which may be due to the dysplasia of cancer cells. Thus, the safety of MSC application in patients bared lung cancer remains to be evaluated carefully.

Lung cancer is a heterogeneous disease with a wide range of clinical pathological features.^79^ The most commonly used transgenic lung cancer models are mice carrying human-derived mutated *EGFR* or *KRAS*. Compared with mice with mutated *hKRAS*, the *EGFR (Del19)* mice showed a much lower cancer burden and a longer process from initiation to early development of adenocarcinoma,^80,81^ which is conducive to observing the occurrence of carcinoma and evaluating the safety of MSCs in tumorigenesis. In our study, the safety of UC-MSCs was investigated by repeatedly transplanting UC-MSCs during the induction of lung adenocarcinoma in EC mice. The time points at which we detected the early precancerous (AAH), the dysplasia of BAC, and the formation of solid micro-adenocarcinoma showed no difference between the MSC and PBS groups. Generally, these results suggest intravascular transplantation of UC-MSCs is safe for the lung. Recently, some groups have used a new xenograft model, as the transplantation of cancer cells and immune cells with or without MSCs, to evaluate the effect in anticancer therapy,^82^ which provides another strategy for modeling by humanizing the immune system with immune cell xenografts. Considering the possible long-distance and long-term regulatory effect of MSCs, to further verify the safety of MSCs in stem cell-based therapy, multi-source MSCs need to be investigated in diverse models as unique TMEs and immune response are associated with different cancers.

## Conclusion

In our study, we used a xenograft model of UC-MSCs by repeatedly injecting MSCs intravascularly into the inducible lung cancer mice that had normal immune system. The initiation and early development of lung adenocarcinoma were not difference between the mice injected with MSCs and PBS. The immune microenvironment in the lungs was not tending more malignant in the MSC group. Briefly, our study provides reliable evidence for the safe of intravenous transplantation of UC-MSCs.

## Supplementary Material

szae065_suppl_Supplementary_Material

## Data Availability

The data underlying this article are available in the article and in its online supplementary material.
