# Supplementary material for: Repeated intravenous transplantation of human umbilical cord mesenchymal stem cells does not promote tumorigenesis in EGFR-mutated lung cancer mice
Source: Stem Cells Transl Med. 2025 Sep 2;14(8):szae065. doi: 10.1093/stcltm/szae065 (PMC12403704; doi:10.1093/stcltm/szae065)
Supplement: szae065_suppl_Supplementary_Material [file szae065_suppl_supplementary_material.pdf]

Supplementary Fig.1

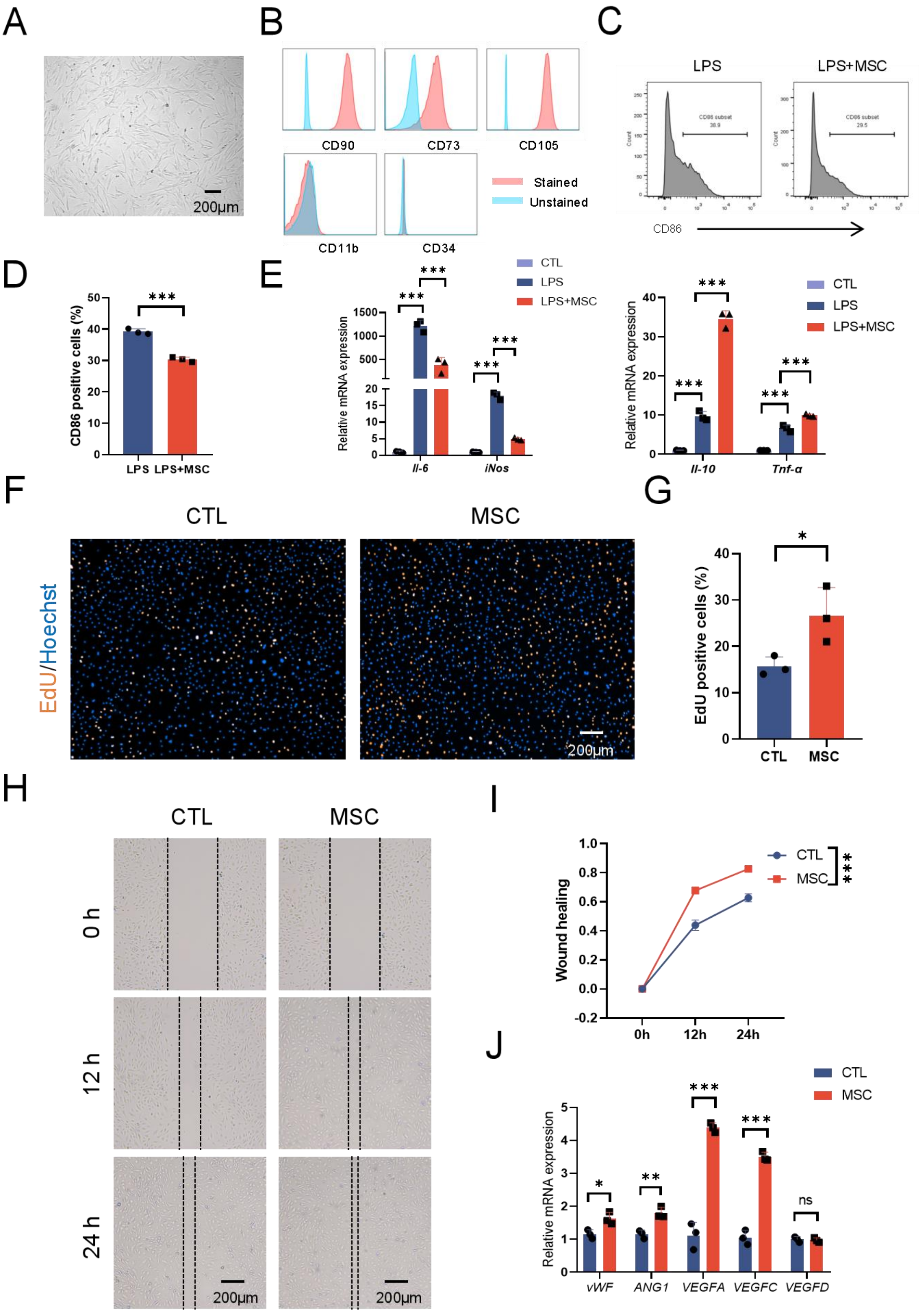

Fig. S1 Identification of UC-MSCs. (A) Morphology of UC-MSCs under *in vitro* culture conditions, scale bar=200μm. (B) Analysis of the surface markers of UC-MSCs by FACS. The UC-MSCs were CD90, CD73, CD105 positive, while lack of CD11b and CD34. (C) Representative images of FACS, RAW264.7 cells were stimulated with LPS for 48h. (D) Statistical analysis of CD86 positive cells in (C), Data are presented as mean±SD, n=3 for each group, t-test. (E) Relative expression of the inflammatory factors when UC-MSCs co-culture with RAW264.7 for 48 h, Data are presented as mean±SD, n=3 for each group, t-test. (F) Representative images of EdU staining when UC-MSCs co-culture with HUVEC cells for 48 h, scale bar=200μm. (G) Statistical analysis of EdU positive cells in (F), Data are presented as mean±SD, n=3 for each group, t-test. (H) Representative images wound-healing assay showing the migratory abilities when UC-MSCs co-culture with HUVEC cells for 48 h, scale bar=200μm. (I) Statistical analysisof wound-healing assay at 24 h, Data are presented as mean±SD, n=3 for each group, two-way ANOVA. (J) Relative expression of the angiogenesis-related factors when UC-MSCs co-culture with HUVEC cells for 48 h, Data are presented as mean±SD, n=3 for each group, t-test. *P < 0.05, **P < 0.01, ***P < 0.001.

Supplementary Fig.2

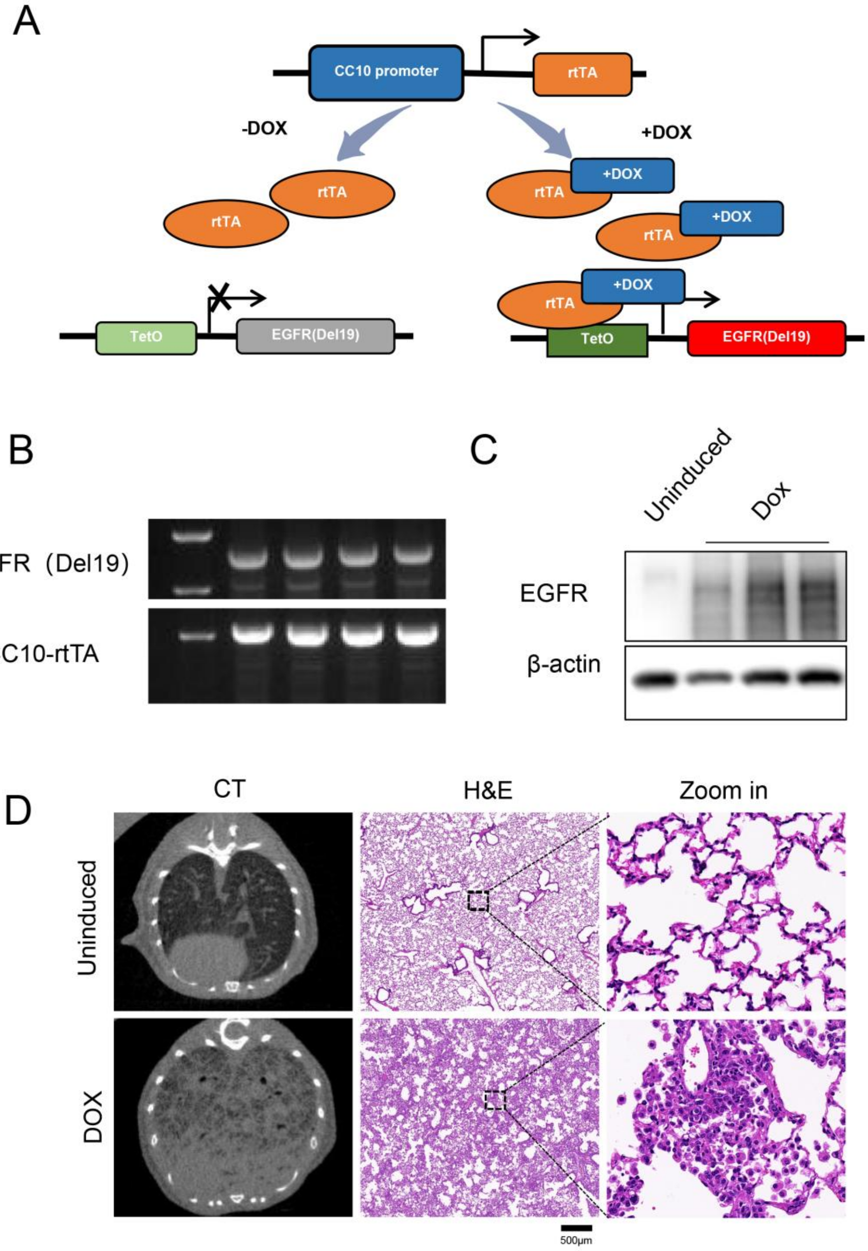

Fig. S2 Induction of lung carcinoma in *TetO-EGFR (Del19);CC10-rtTA* mice (designated EC mice). (A) Schematic of carcinoma induction in EC mice. (B) Genotype identification of EC mice. (C) EGFR protein expression in lung were detected by western blot in EC mice, with and without DOX diet induced. (D) CT scans and H&E staining of EC mice, with and without DOX diet induction, after 4 weeks. The early precancerous atypical adenomatous hyperplasia appears when induced with DOX diet, scale bar=500μm.

Supplementary Fig.3

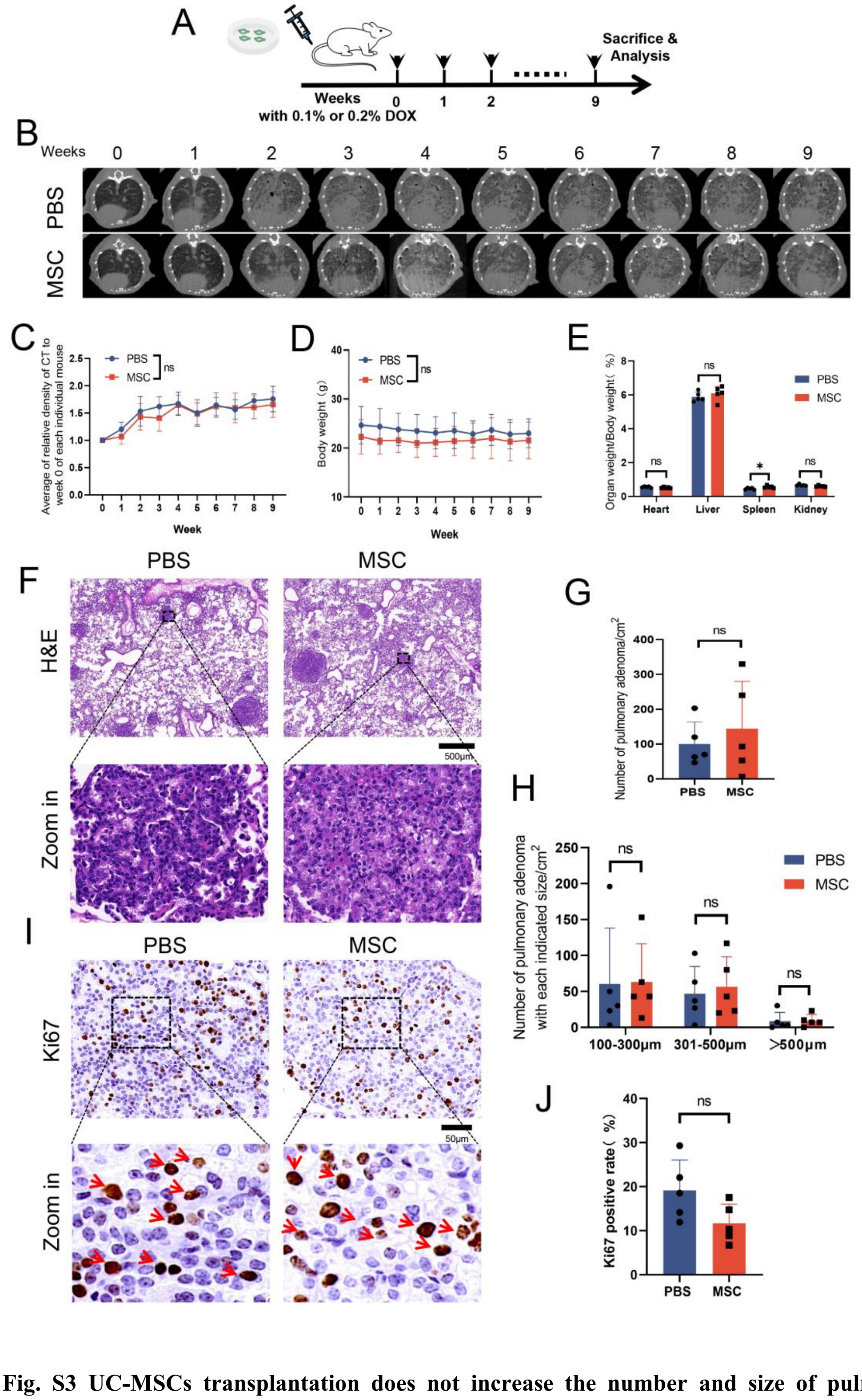

Fig. S3 UC-MSCs transplantation does not increase the number and size of pulmonary adenomas compared with the PBS group. (A) Schematic of EC mice induction to form solid micro-adenomas after 9 weeks. Mice were fed with either a 0.1% or 0.2% DOX diet, with weekly transplantation of UC-MSCs. PBS was injected as a control. CT scans and weight were recorded weekly. EC mice were sacrificed after 9 weeks when the adenocarcinoma revealed solid features as

previously observed. (B) Representative images of CT scans at different time points. (C) Grayscale analysis of CT images from (B). The relative grayscale of selected regions in the CT images was normalized to week 0. Data are presented as mean±SD, n=5 for each group, two-way ANOVA. (D) Body weight of mice in the MSC and PBS groups. Data are presented as mean±SD, n=5 for each group, two-way ANOVA. (E) Statistical analysis of mice heart, liver, spleen, and kidney weight relative to body weight. Data are presented as mean±SD, n=5 for each group, t-test. (F) Representative images of H&E stained lung sections after 9 weeks, scale bar=500μm. (G) Statistical analysis of tumor number in (F). Data are presented as mean±SD, n=5 for each group, t-test. (H) Statistical analysis of the number of tumors of different sizes in (F). Data are presented as mean±SD, n=5 for each group, t-test. (I) Representative images of IHC with Ki67 staining of lung sections after 9 weeks of induction. Ki67-positive cells are indicated by red arrows in the enlarged images, scale bar=50μm. (J) Statistical analysis of Ki67-positive cells in (I). Data are presented as mean±SD, n=5 for each group, t-test. *$P<0.05$.

Supplementary Fig.4

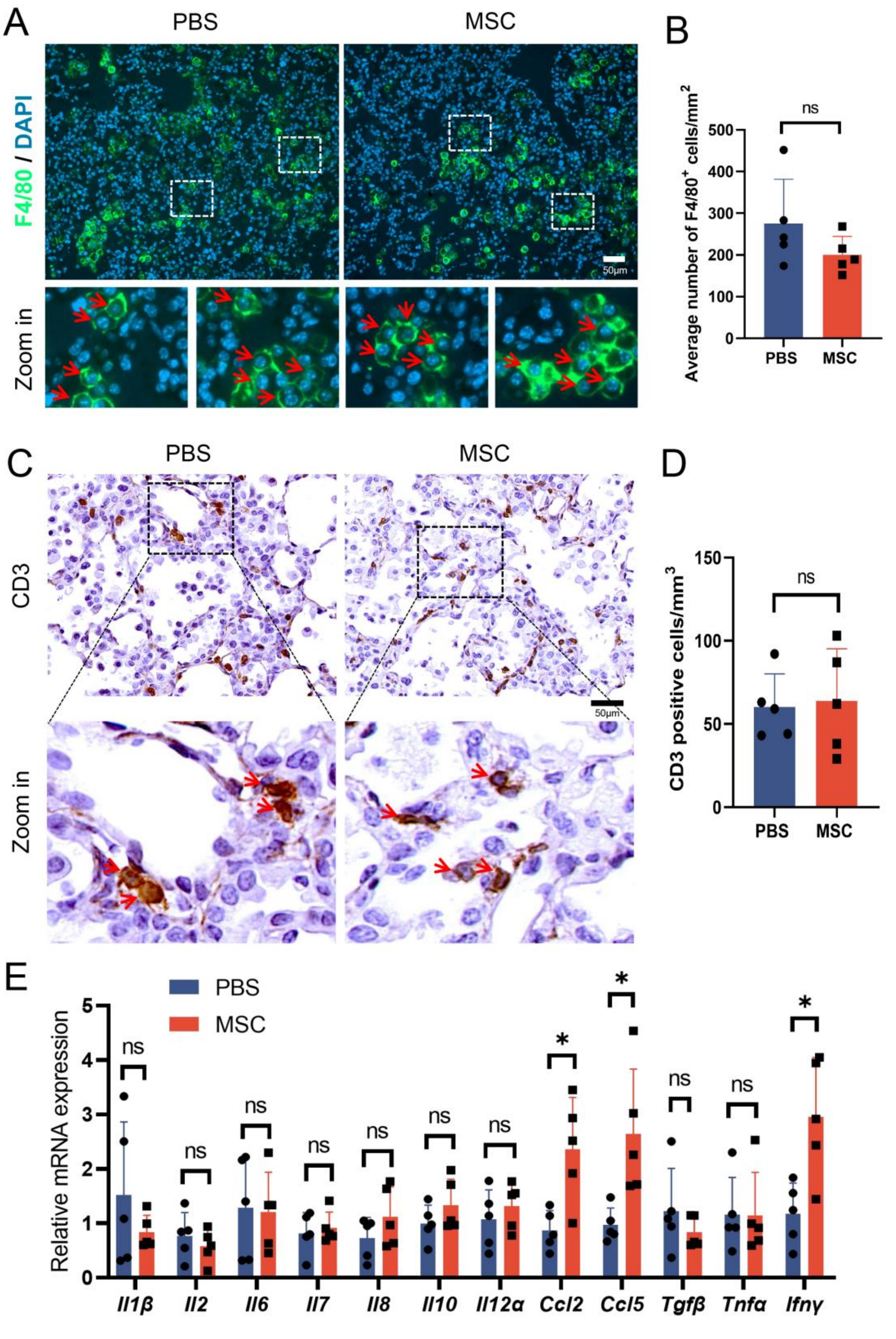

Fig. S4 The local immune microenvironment shows minor differences in the formation of pulmonary adenomas between the MSC and PBS groups. (A) Representative images of IF with F4/80 staining in lung tissue of EC mice 9 weeks post-induction with 0.2% DOX diet. F4/80-positive cells are indicated by red arrows in the enlarged images, scale bar=50μm. (B) Statistical analysis of F4/80-positive cells in (A). Data are presented as mean ± SD, n=5 for each group, t-test. (C)

Representative images of IHC with CD3 staining for lung tissue 9 weeks post-induction with 0.2% DOX diet. CD3-positive cells are indicated by red arrows in the enlarged images, scale bar=50μm. (D) Statistical analysis of CD3-positive cells in (C). Data are presented as mean ± SD, n=5 for each group, t-test. (E) Relative expression of the indicated gene in lung tissue from the MSC and PBS groups 9 weeks post-induction. Data are presented as mean ± SD, n=5 for each group, t-test. *P < 0.05.

Supplementary Fig.5

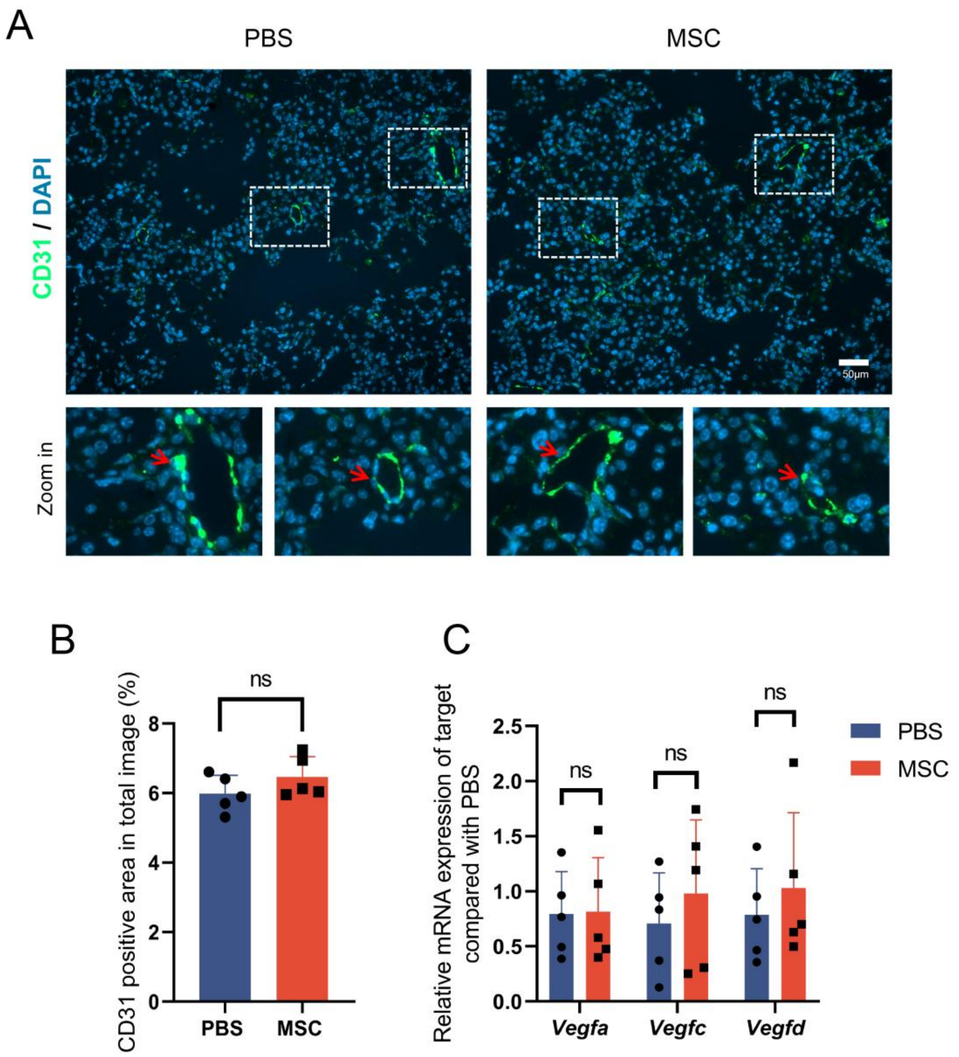

Fig. S5 No difference in angiogenesis after the formation of solid-feature lung adenocarcinomas in the MSC and PBS groups. (A) Representative images of IF with CD31 staining for lung tissue 9 weeks post-induction with 0.2% DOX diet. CD31-positive areas are indicated by red arrows in the enlarged images, scale bar=50μm. (B) Statistical analysis of the positive area in (A). Data are presented as mean±SD, n=5 for each group, t-test. (C) Relative expression of the indicated gene in lung tissue from the MSCs and PBS groups 9 weeks post-induction. Data are presented as mean±SD, n=5 for each group, t-test.

Supplementary Fig.6

A

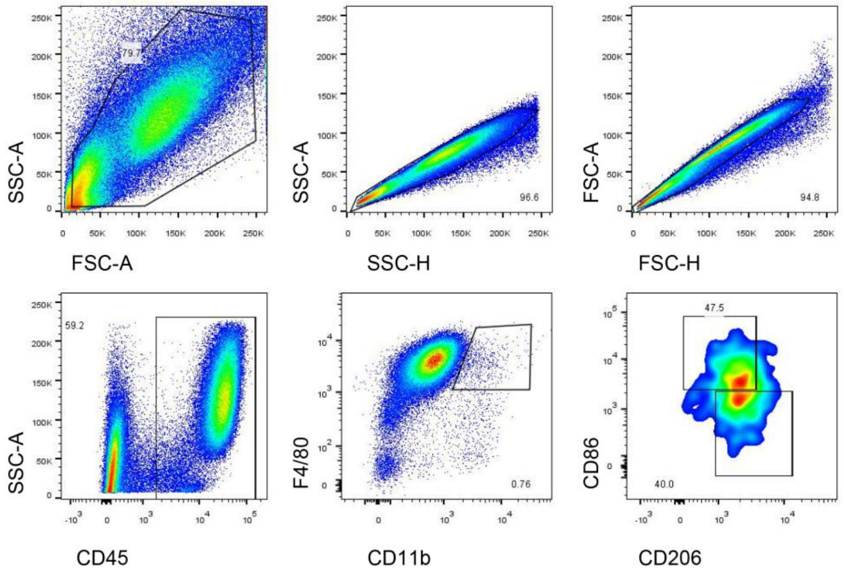

Fig. S6 Gating strategy of the macrophages by FACS. (A) Gating strategy for analyzing macrophages in the cells suspension of lung tissue. After gating the single cell (by SSC-A/H and FSC-A/H), macrophage(F4/80^{high}, CD11b^{-}) cells were analyzed by gating on the CD45^{+} population, and M1(CD86) or M2(CD206) were analyzed by gating on the F4/80^{high} and CD11b^{-} population.

Supplementary Fig.7

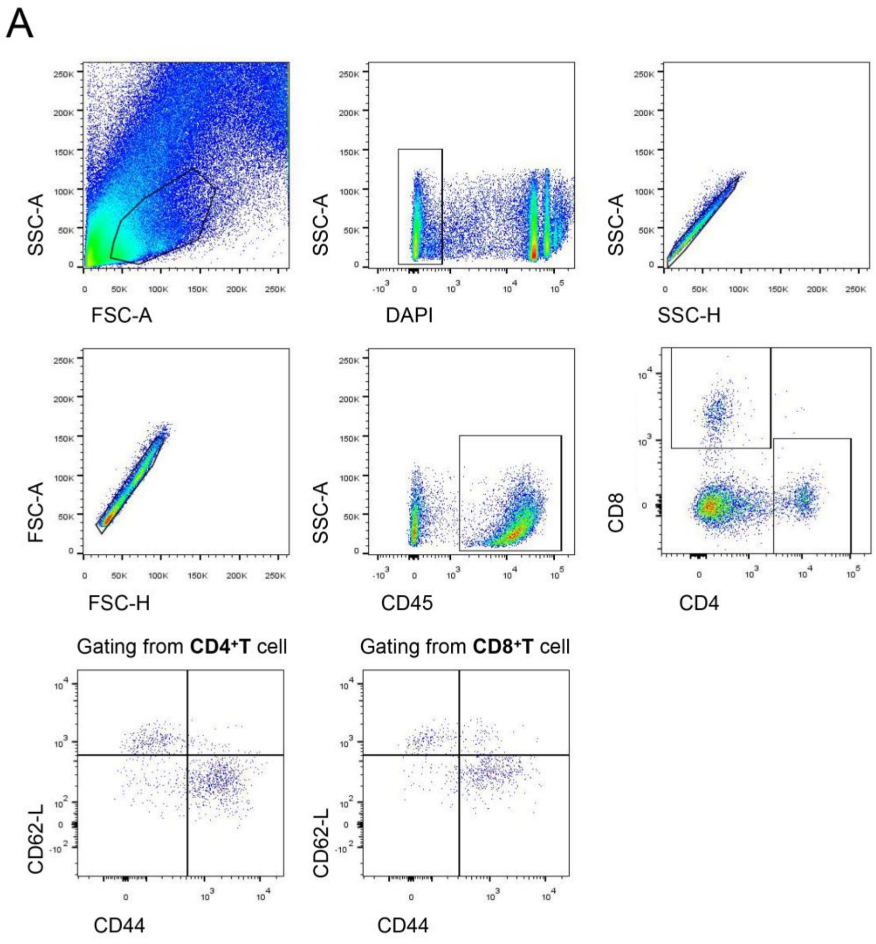

Fig. S7 Gating strategy of the T cells by FACS. (A) Gating strategy for analyzing T cells in the cell suspension of lung tissue. After gating the live cells (DAPI$^-$) and single cells (by SSC-A/H and FSC-A/H), CD4 or CD8 T cells were analyzed by gating on the CD45$^+$ population. The effector T cells, memory T cells and naïve T cells were also analyzed by gating on the CD45$^+$ population.

SUPPLEMENTARY MATERUALS AND METHODS

1 Western blotting for the detection of EGFR

To detect the expression of the EGFR protein in the lungs, approximately 10mg of tissue was homogenized (Jingxin, JXFSTPRP-48) in 200μL of RIPA lysis buffer (Beyotime, P0023A). The homogenate was centrifuged at 12000g for 5 minutes at 4°C, and the supernatant was collected and mixed with SDS loading buffer for sample preparation. Tissue samples (approximately 10μL) were loaded for electrophoresis by SDS-PAGE and transferred to PVDF membranes (BioRad). After blocking with 5% BSA (Sigma, B2064-100G), the membranes were incubated overnight with primary anti-mouse EGFR antibody (Proteintech, 18986-1-AP, 1:2500) and anti-mouse β-actin antibody (ABclonal, AC026, 1:5000) at 4°C. The next day, the membranes were thoroughly washed and incubated with horseradish peroxidase-conjugated anti-rabbit (Cell Signaling, 7074, 1:5000) for 1 hour at room temperature. Protein signals were then detected using an enhanced chemiluminescence kit (BioRad).

2 Real-time quantitative polymerase chain reaction

Total RNA is extracted from approximately 10mg of lung tissue and dissolved in 50μL of ddH2O. The concentration is then determined using a NanoDrop (Thermo). The RNA is subsequently reverse-transcribed (Vazyme), and real-time PCR is performed on a QuantStudio 6 Flex system (Applied Biosystems) with a 2×Taq mix (Vazyme). The amount of target mRNA is normalized to that of β-actin, and gene expression quantities are determined according to the relative Ct. The qPCR primer motif can be found in Supplementary Table 1.

3 In vivo imaging system (IVIS)

We obtained UC-MSCs carrying luciferase (luci-MSCs) through lentivirus infection. 0.5 million luci-MSCs were slowly transplanted through the tail vein. At 2h or 24h after transplantation, 200μL of D-luciferin (15mg/mL) (Solarbio, 115144-35-9) was injected intraperitoneally. To prevent interference from mouse hair during signal acquisition, the abdominal hair of the mouse was removed. *In vivo* imaging was then performed using a PerkinElmer IVIS Lumina III after isoflurane anesthesia.

4 Flow Cytometry

UC-MSCs surface markers were detected with CD34, CD11b, CD105, CD90, and CD73. UC-MSCs in culture dishes were digested and collected. The cells were washed with PBS and divided into 1×10^6 cells per 1.5mL tube for flow cytometry antibody staining. For the analysis of immune cells in lung tissue, the lung tissues were isolated and ground on a 200 mesh. We collected the cell suspension and lysis solution (Biolegend, B350185) at low temperature. Approximately 3×10^6 cells from each sample were divided into 1.5mL tubes for flow cytometry antibody staining. For T cell analysis, we used anti-CD4, anti-CD62L, anti-CD44, anti-CD45, and anti-CD8 for staining. For macrophage cell analysis, we used anti-F4/80, anti-CD206, anti-CD86, anti-CD45, and anti-CD11b for staining. For intracellular staining of anti-CD206, cells were fixed and permeabilized using Cytofix/Cytoperm (BD, 1152330). The Flow Cytometry antibody list can be found in Supplementary Table 2.

All of the above antibody staining was performed at 4°C in the dark. Staining was terminated with PBSF (PBS containing 2% FBS) and washed. The cells were resuspended in PBSF containing 0.2mg/mL DAPI and passed through a 300 mesh before being analyzed by flow cytometry (Becton Dickinson). Data were analyzed using FlowJo software (FlowJo V10).

5 Functional Identification of UC-MSCs

RAW264.7 (1×10^5 cells) were co-cultured with UC-MSCs (3×10^5 cells) and seeded into six-well plates. After overnight incubation for cell adherence, 200 ng/mL LPS was added and further cultured for 48 hours. Cells were stained with anti-CD11b and CD86 and collected by flow cytometric analysis, following the analysis of inflammatory factor with Q-PCR.

UC-MSCs (2×10^5 cells/well in upper chamber) and HUVEC(1.8×10^5 cells/well in bottom chamber) were co-cultured in transwell (0.4μm pore-sized filters, 6-well culture plates). For EdU assay, Edu was labeling for 8h after 48 hours co-culture, and than performed with BeyoClick™ EdU-555 kit (C0075S). Fluorescence images were captured by fluorescence microscope (ZEISS). For scratch wound-healing assay, after 24 hours of co-culture, monolayer of HUVEC was scratched with a 200μL pipette tip after the cells had been attached. Washed with PBS to remove floating cells. At 0 h, 12 h and 24 h, the sample location of was photographed. The migration area rate was determined as the closure area to initial wound ratio.

5 Statistical Analysis

All experiments were conducted at least three times. Statistical analysis was performed using GraphPad Prism 8.0.2. Results are presented as the mean ± standard deviation (SD). For data that followed a Gaussian distribution, an unpaired two-tailed Student's t-test or two-way ANOVA are used to determine statistical significance. For data that did not follow a Gaussian distribution, the Mann-Whitney test was used. (ns = not significant, * $P < 0.05$, ** $P < 0.01$, *** $P < 0.001$).
